# A neuroscience perspective on the plasticity of the social and relational brain

**DOI:** 10.1111/nyas.15319

**Published:** 2025-04-03

**Authors:** Tania Singer

**Affiliations:** ^1^ Social Neuroscience Lab Max Planck Society Berlin Germany

**Keywords:** brain plasticity, compassion, dyads, empathy, mental training, social neuroscience, theory of mind

## Abstract

Over the past two decades, the fields of social and contemplative neurosciences have made significant strides. Initial research utilizing fMRI identified neuronal networks involved in empathy, mentalizing, and compassion, as well as complex interactions among these networks. Subsequent studies shifted to testing the plasticity of these social skills via different types of mindfulness‐ or compassion‐based mental training programs, demonstrating brain plasticity, enhanced social capacities and motivation, as well as improved mental health and overall well‐being. Next, researchers developed scalable evidence‐based online mental training programs to address the growing levels of mental health problems and loneliness, both exacerbated by the COVID‐19 pandemic. Innovative approaches, such as novel relational partner–based practices and online app–based dyadic training programs, offer scalable solutions to counteract ongoing societal and mental health deterioration. Current studies are now applying the above findings to support resilience building within diverse domains of society and professional populations—such as healthcare workers and teachers—at high risk of burn‐out. Future research should explore the broader impact of such training‐related individual changes on larger systems, potentially leading to the development of a *translational social neuroscience* approach that leverages insights from social brain plasticity research to support societal needs, thereby enhancing resilience, mental health, and social cohesion.

## Graphical abstract

This review discusses both the psychological and neuroscientific foundations of human social brains that support social emotions, such as empathy and compassion, and socio‐cognitive capacities, such as theory of mind.

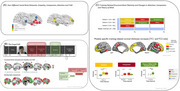

## INTRODUCTION

Our world is facing diverse problems and crises ranging from increasing mental health problems, social isolation, and loneliness—all exacerbated by the worldwide COVID‐19 pandemic, polarization, and loss of social cohesion in societies. In the face of such poly‐crises, societies are in urgent need of innovative solutions that foster mental health, resilience, and social cohesion and reconnect people with themselves and each other across national borders and social divides. Humans are inherently social beings equipped with social capacities that allow sharing and understanding of each other's feelings, thoughts, and needs; in addition, humans have the ability through life experience and active practice to extend circles of tolerance, compassion, and inclusivity needed to develop a sense of shared humanity.

Recent advances in social and contemplative neurosciences have contributed to a better understanding of how the social brain works and how social capacities such as empathy, compassion, or cognitive perspective–taking can be trained by making use of different types of contemplative mental training interventions targeting specific socio‐affective or cognitive skills.^1^, ^2^, ^3^, ^4^, ^5^, ^6^ These meditation‐, mindfulness‐, or compassion‐based secular mental training interventions—often called mindfulness‐based interventions (MBIs)—have further proven to be efficient at boosting mental health,^7^, ^8^, ^9^ reducing stress,^2^, ^10^, ^11^ and increasing well‐being^1^, ^12^, ^13^ and human prosociality^14^, ^15^, ^16^ and are therefore promising tools for addressing some of the most urgent societal problems.

This review aims to give an overview of both the psychological and neuroscientific foundations of our social brains supporting social emotions such as empathy and compassion and socio‐cognitive capacities such as theory of mind (ToM). After defining these terms, I describe the underlying neuronal circuitries and their interaction.

In the second section, I illustrate recent advances in how to train social capacities with the help of contemplative mental training interventions. I will review the associated positive benefits of such mental training on mental health and resilience, focusing on concrete study examples of three outstanding long‐term mental training studies: the Shamata project,^17^ the MeditAging study,^18^ and the ReSource project.^19^ The latter is a 9‐month large‐scale longitudinal mental training study that illustrates that *it matters what you practice*, in that each type of practice has its specific fingerprint on diverse outcome measures ranging from brain to hormones to behavioral markers.^20^

In the third section, I move from the social brain to the relational brain by introducing a specific type of novel and highly promising mental practice done daily together with a partner over several weeks as a skill‐learning approach, the so‐called contemplative dyads.^21^ To show the specific benefits of such relational practice in comparison to mental practices done alone, I then summarize recent findings from a large‐scale study of my research group, the CovSocial project,^22^ directly comparing a 10‐week app‐based classic mindfulness program with such novel socio‐emotional relational dyadic programs. I also propose new mechanistic models based on first findings revealing different underlying mechanisms of these different practice types, enabling the field to move toward a more individual‐based approach to mental health and appropriate interventions.

Finally, I discuss how dyadic social intervention approaches can lead to scalable online programs that in turn directly address societal problems, such as the drastically increasing levels of loneliness, polarization, and societal disconnects.^23^, ^24^, ^25^ I conclude with ideas for future directions that social and contemplative neuroscience could take and advocate for a translational social neuroscience approach to benefit society.

## DISSECTING THE SOCIAL BRAIN

With the emergence of the field of social neuroscience around the turn of the century,^26^, ^27^, ^28^, ^29^, ^30^, ^31^ neuroscientists and psychologists started to investigate how our brains make sense of our social realities and allow people to understand and interact with each other (for reviews, see Refs. 27, 32, 33). Researchers began to dissect the social brain and identify the different underlying neural networks that drive different social capacities. Early investigations of how social and emotional cues are encoded in the brain explored the neural processing of facial emotional expressions,^34^, ^35^ as well as neural mechanisms underlying cognitive perspective‐taking, the ability of ToM, or mentalizing.^36^, ^37^, ^38^, ^39^, ^40^

In parallel, the discovery of mirror neurons in macaque brains which fire not only if the monkey performs a specific action but also when the animal sees the experimenter performing the same action, indicated that the brain understands the action goals and intentions of others by simulation, that is, by activating the neural representations processing our own actions.^41^, ^42^, ^43^, ^44^ This discovery was then quickly extended to the domain of thoughts and emotions^45^ in suggesting that to understand the emotions of others we activate neuronal networks processing these emotions in ourselves. And indeed, imaging studies in humans revealed that shared networks in our brains explained our ability to empathize with other's emotions^46^, ^47^, ^48^ followed by compassion.^49^, ^50^, ^51^ The study of the neuronal basis of compassion and its differentiation from the biological underpinnings of empathy^51^ only emerged with the birth of a new field, the field of contemplative sciences,^52^, ^53^, ^54^, ^55^ with its secular focus on how secular meditation‐ and mindfulness‐based training programs can improve mental and physical health by fostering social competencies and skills such as mindfulness, perspective‐taking, empathy, and compassion.^20^, ^51^, ^56^, ^57^, ^58^, ^59^

Before diving into depth into how the social brain can be trained, I will first define the most important concepts discussed below and give an overview of the neural circuitries underlying our abilities to feel with (empathy) and for (compassion) and to understand the thoughts of other people (ToM).

### Definition and distinctions of key concepts

In attempting to understand the dimensions and functions of our social brain, particularly our capacities for empathy, compassion, and mentalizing, it is necessary to first define these constructs. However, the literature from clinical psychology, philosophy, and the social sciences reveals no single definition of these terms.^47^, ^51^, ^60^ Taking a pragmatic approach, I propose here a set of widely used definitions in social neuroscience and psychology as the basis for the present review and research focusing on the social brain and its plasticity.

*Empathy*. As for all terms used in the present review, there is no single definition for empathy either.^61^, ^62^, ^63^, ^64^, ^65^, ^66^, ^67^ To encourage alignment in social neuroscience and psychology, de Vignemont and Singer^47^ proposed a comprehensive definition: Empathy entails four components: (1) experiencing an affective state, (2) similar to that observed or imagined in another person, (3) elicited through observing or imagining the other's affective experience, and (4) knowing that the source of this state was evoked by the other, that is, having a clear self–other distinction (in contrast to emotion contagion). Dan Batson simplified this as *feeling with* another person, comparable to emotional resonance.^63^

*Empathic distress* (or personal distress) occurs when the empathic response to another's suffering becomes so intense that the healthy self–other distinction blurs and the other's suffering becomes one's own burden. It involves feelings of anxiety, discomfort, or stress that arise from the perceived pain or discomfort of another individual. Such overidentification with the other's suffering leads to personal distress, which often results in a desire to socially disengage.^68^, ^69^

*Emotional contagion* involves sharing emotions with another, however, without the self–other distinction present in empathy.^47^, ^70^ It is seen as a precursor to empathy, observed in young children and also in many animals.^46^, ^71^, ^72^ It operates in domains such as pupillary contagion, temperature contagion, stress contagion at the hormonal level, and emotion contagion.^73^, ^74^, ^75^, ^76^, ^77^

*Compassion* (empathic concern^78^, ^79^ or sympathy^65^, ^80^, ^81^) entails feeling for another person^51^, ^63^, ^82^ and invokes rather feelings of sorrow or concern for others who are suffering^83^ but not feeling the same emotion as the other. Compassion, according to Keltner and Goetz, can be defined as “the emotion one experiences when feeling concern for another's suffering and desiring to enhance that individual's welfare.”^84^ Compassion is viewed as an altruistic motivation rooted in evolutionary systems of care and affiliation^51^, ^85^, ^86^, ^87^, ^88^ and involves a desire to help alleviate others’ suffering.^51^, ^87^, ^89^, ^90^ Such altruistic motivation can be accompanied by feelings of concern, warmth, and love and subsequently can drive taking action to alleviate suffering.^91^, ^92^, ^93^, ^94^, ^95^, ^96^

*ToM* (or mentalizing, or cognitive perspective‐taking) contrasts with socio‐emotional capacities and refers to the cognitive ability to understand and attribute mental states to others—such as beliefs, desires, intentions, and emotions. It involves recognizing that others have thoughts, feelings, and perspectives that may differ from one's own, and that these mental states influence behavior.^36^, ^37^, ^38^, ^40^, ^97^, ^98^, ^99^

### Differential brain networks for ToM and empathy

After defining relevant concepts, I will delineate the neuronal networks underlying these different social capacities, starting with the oldest discovery of social brain networks subserving our ability to mentalize (or ToM), followed by empathy research. As the distinction between the biological basis of empathy and compassion is tied to the emergence of contemplative neuroscience and the training of compassion, I will summarize the neuronal networks underlying compassion when introducing the topic of social brain plasticity.

#### Neural signatures of ToM

The investigation of the neuronal networks underlying our ability to mentalize was initially discussed and investigated by philosophy,^97^, ^98^ developmental psychology, clinical psychology,^100^ and primatology.^36^, ^101^ It was suggested that understanding other people's minds is a special cognitive ability that develops late in human ontogeny, may be deficient in autistic children,^40^, ^102^, ^103^ and is supported by specialized neuronal circuitries geared to understanding the intentions, beliefs, and thoughts of others.^104^

With new non‐invasive imaging technologies being developed, behavioral research on ToM extended into cognitive neuroscience. Typically, stories, cartoons, or animated shapes were used to test participants’ ability to infer attributes about others’ minds versus physical properties of the world.^105^, ^106^, ^107^, ^108^, ^109^, ^110^, ^111^ When the field of neuroeconomics emerged, researchers also developed mentalizing experiments comparing brain activation when participants played strategic monetary games against intentional versus non‐intentional players.^48^, ^112^, ^113^

**FIGURE 1:**
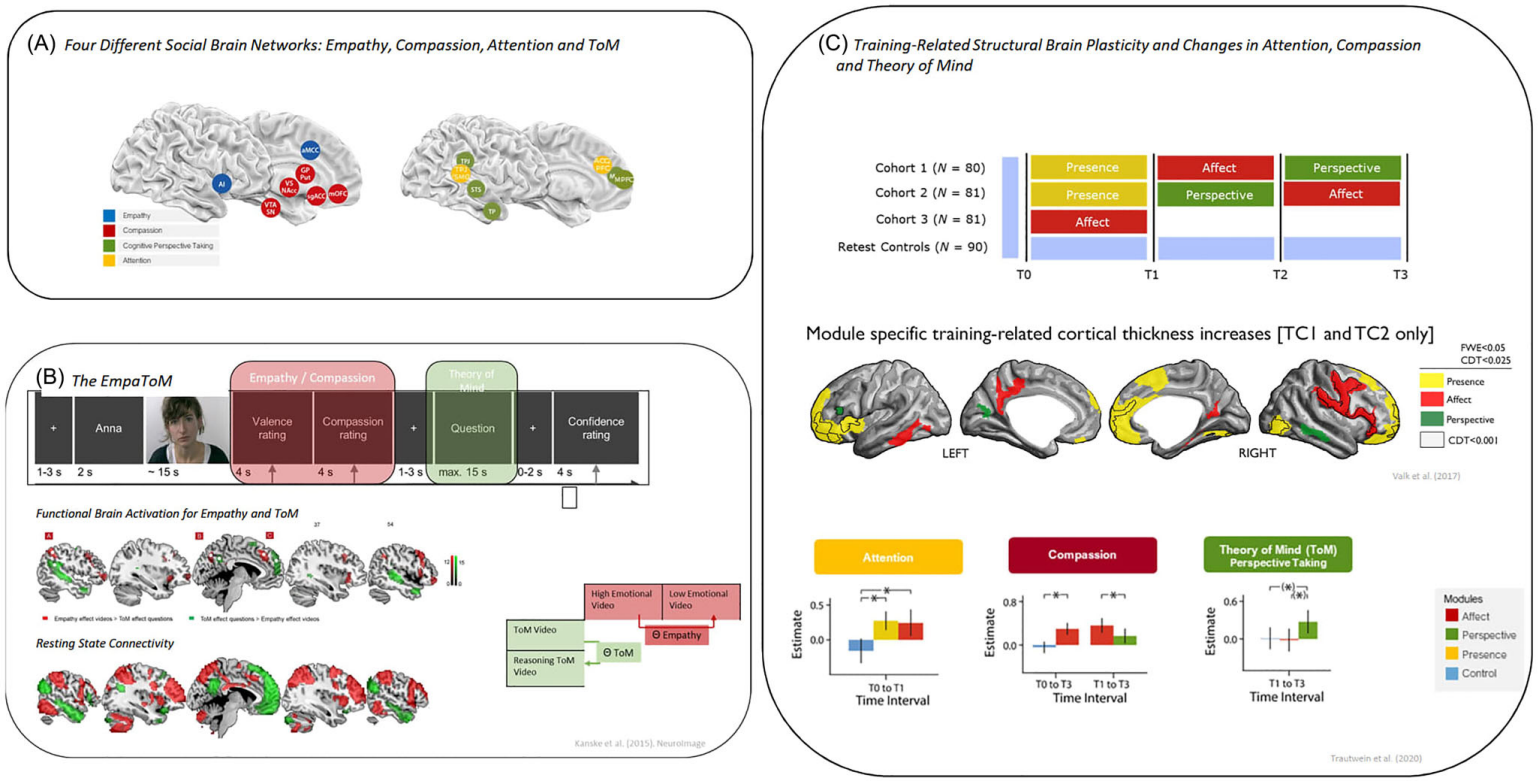
This figure depicts different brain circuitries underlying our capacity for attention, empathy, compassion, and theory of mind (ToM) and its plasticity. Panel A depicts a schematic overview of four brain networks underlying our capacity for empathy (blue), compassion (red), attention (yellow), and ToM (green). Panel B illustrates results from the EmpaToM task designed to assess both empathy and ToM (and compassion) within a single task using video materials and different types of ratings. The seeds for resting state connectivity are displayed as white dots in the middle figure. Although contrasting high versus low emotional videos results in functional activations in empathy networks (in red), the contrast between ToM videos as compared to equally difficult reasoning videos shows the so‐called ToM or mentalizing network (green); for details, see Kanske et al.^118^ Panel C depicts differential findings from the ReSource project after a 3‐month attention‐based training module (presence—yellow), a 3‐month socio‐emotional care‐based training module (affect—red), and a 3‐month socio‐cognitive perspective‐taking training module (perspective—green) on gray matter plasticity in the brain (for details, see Ref. 4) as well as associated behavioral changes in attention (as measured through a cue‐flanker task), compassion, and ToM (as measured through the EmpaToM; for details, see Ref. 3).

Several meta‐analyses reveal key brain areas activated when mentalizing: ventral temporoparietal junction (TPJ), superior temporal sulcus, temporal poles, medial prefrontal cortex (mPFC), and precuneus/posterior cingulate.^114^, ^115^, ^116^, ^117^, ^118^, ^119^, ^120^ This so‐called basic ToM or mentalizing network is illustrated schematically in green in Figure 1A. There is ongoing discussion about the exact functions and underlying computational processes subserved by the single‐brain regions of this large‐scale network, with some researchers postulating a special role of rTPJ in making inferences about mental states and beliefs,^110^ others postulating a more general role of this area in self–other distinction,^121^ or even more general attentional or multi‐sensory integration processes.^109^, ^120^, ^122^ Similarly, several researchers have postulated a special role of mPFC in attributing thoughts, intentions, and beliefs to others, but also when reflecting on mental states of the self^123^ or when mind wandering or daydreaming.^124^

#### Shared neuronal networks in empathy

Inspired by mirror neuron research^41^, ^42^, ^43^, ^44^ and the principle of understanding others’ actions via simulation, social neuroscientists began investigating empathy by comparing brain responses elicited by experiencing certain emotions or sensations versus observing others’ experiences (for a review, see Ref. 32). Early fMRI studies on empathy revealed shared neuronal representations for both first‐hand and vicarious experiences of emotions such as disgust,^125^ pain,^48^, ^126^ pleasant and unpleasant tastes,^127^ and neutral, pleasant, and unpleasant touch.^128^, ^129^ These studies confirmed that indeed the principle of shared networks was true in that we seem to share the emotions of others by activating those brain networks that subserve the processing of the very same emotional states in ourselves. By consequence, there is no single empathy area or network, as the neuronal circuitry activated will depend on the specific type of the shared emotion.

**FIGURE 2:**
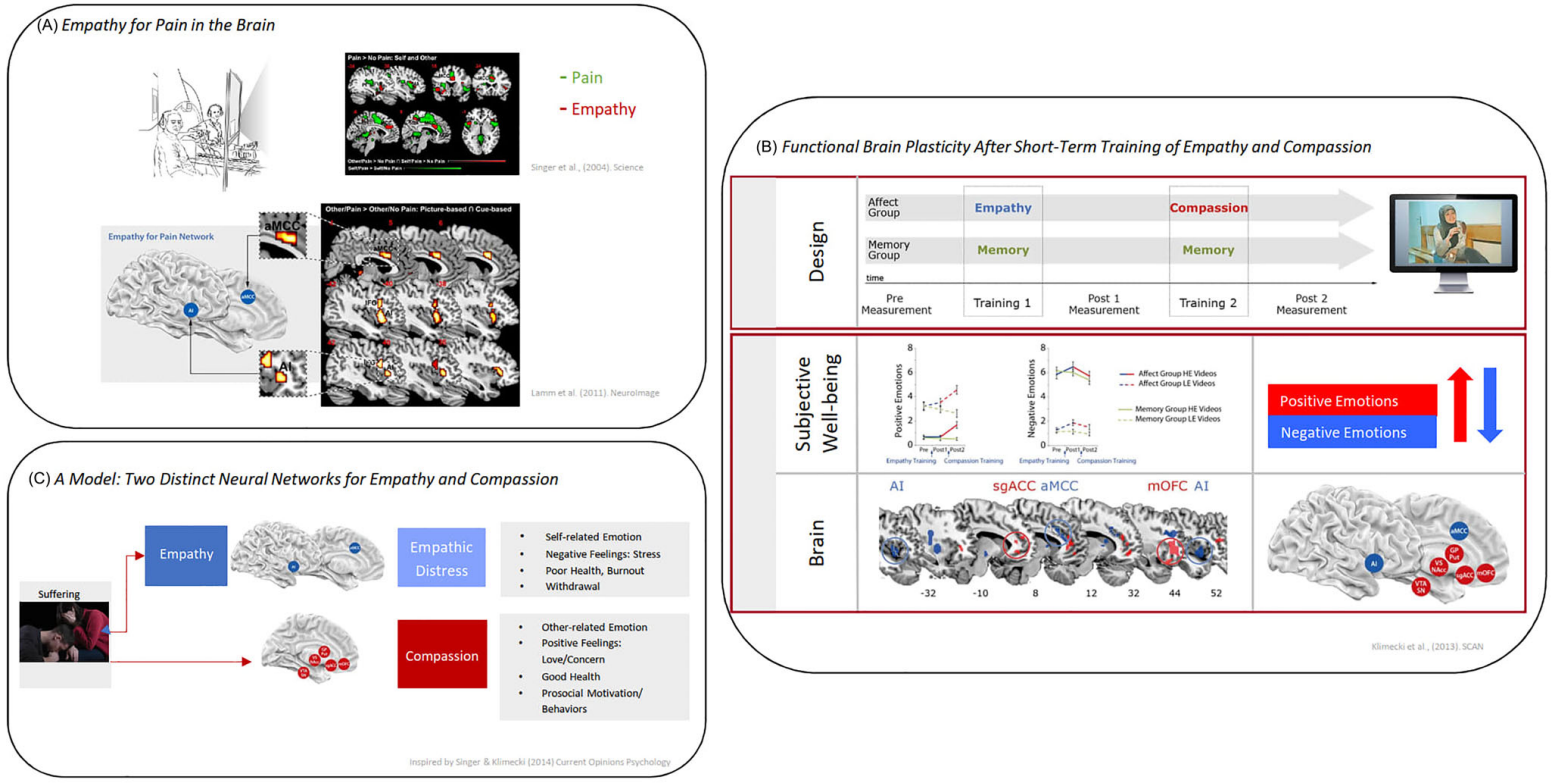
This figure depicts the neural circuitries underlying our capacities for empathy and compassion. Panel A displays the empathy for pain paradigm (for details, see Refs. 48 and 135), enabling the assessment of the networks activating if a scanned subject receives painful stimuli to her/his hand (brain activations in green) and when only knowing that another person outside of the scanner is suffering pain (brain activations in red).^48^ The lower picture illustrates results from a meta‐analysis^130^ performed on many empathy‐for‐pain studies showing the typical empathy‐for‐pain core‐network activated with foci in anterior insula (AI) and anterior middle cingulate cortex (aMCC). Panel B illustrates the results of a functional brain plasticity study,^90^ in which participants first trained in empathy and then in compassion (and an active control group in a mnemonic technique). Functional brain activations as well as affect ratings were assessed pre‐post‐training while watching high or low emotional videos, the SoVT^177^ task. Although empathy training increased negative affect, compassion boosted positive affect and lowered negative affect back to baseline levels. The lower picture shows functional plasticity in empathy‐for‐pain networks (AI, mACC) after empathy training and increased activation in compassion‐related areas such as subgenual anterior cingulate cortex (sgACC) and medium orbito‐frontal cortex (mOFC). Other increased activations after compassion training in areas such as nucleus accumbens (Nacc) and ventral striatum (VS), ventral tegmental area (VTA) and substancia nigra (SN) and globus pallidus (GP) and putamen (Put) are schematically shown in the last figure. Panel C is a schematic illustration of a refined model first proposed by Singer and Klimecki,^51^ suggesting two pathways for how to respond to the suffering of others. Suffering can result in a healthy empathic response, but this can turn into empathic distress if the healthy self‐other distinction vanishes and becomes personal distress. Another pathway of how to respond to the suffering of others is with compassion, which relies on another neuronal circuitry and system.

The majority of empathy studies have been performed in the area of pain,^32^, ^130^, ^131^, ^132^ revealing shared neural activations in the anterior insula (AI) extending into the inferior frontal gyrus (IFG) and anterior middle cingulate cortex (aMCC), part of the pain matrix associated with suffering's emotional experience.^131^, ^132^ By contrast, and as illustrated in Figure 2A (activation of the pain matrix depicted in green), somatosensory and motor components of the pain matrix were not re‐activated, suggesting that we only activate the emotional components of pain, not its sensory aspects.^48^ Meta‐analyses have later identified the AI/IFG and aMCC regions as a core network that is activated whenever we witness the suffering of others (see Figure 2A), whether present in person in the scanner environment as in the original Singer et al. paradigm,^135^ or whether presented only via video, stories, or even induced by imagination.^130^, ^133^, ^134^ A second wave of neuroscientific empathy research started to look at modulatory factors of neuronal responses such as perceived fairness of others^135^ and ingroup–outgroup perception, ^136^ and person‐specific factors such as the level of alexithymia or gender^130^ (for reviews, see Refs. 132 and 137).

#### Interactions between empathy and ToM

Empathizing and mentalizing are distinguishable processes relying on distinct brain circuitries but are often co‐activated during complex social cognition. However, research on empathy and ToM was traditionally separate. To be finally able to study their interaction with one single paradigm in a person (see Figure 1B), my research group developed the EmpaToM task, which measures empathy, compassion, and ToM in a single task, replicating dissociable empathy‐for‐pain (Figure 1B, activation in red) and mentalizing networks (ToM, Figure 1B, activation patterns in green) within a person^118^; this demonstrated that these two networks have distinct functional connectivity to well‐known resting state networks such as the alerting network (with seeds in the empathy network, see Figure 1B) and the default‐mode network (with seeds in the ToM network, see Figure 1B). Similarly, behavioral and neural evidence shows no significant correlation between individual differences in empathy and ToM, indicating distinct abilities, that is, strong empathizers are not necessarily proficient mentalizers and the other way around.^138^

In line with such independent functions, selective impairments in empathy or ToM have been observed in different psychopathologies such as autism and psychopathy. In autism, ToM is deficient,^102^, ^103^, ^139^, ^140^, ^141^ whereas no empathy deficits are observed when controlling for co‐morbidity with alexithymia.^142^, ^143^, ^144^, ^145^ In psychopathy or chronic aggression, in contrast, ToM is intact, but the propensity to empathize with others is reduced.^146^, ^147^, ^148^

Despite being separable, empathy and ToM are jointly required in many complex social situations,^117^, ^149^ and both are necessary for efficient communication and conflict resolution and are therefore often observed to be jointly co‐activated. Most importantly, the existence of such distinct social brain networks has direct implications for what will be the topic of our next section: the trainability and plasticity of the social brain. Thus, it is likely that different types of mental practices need to be devised to induce brain plasticity in these diverse brain networks if we were to increase our capacities to mentalize or empathize.

## PLASTICITY OF THE SOCIAL BRAIN

The discovery of the difference between the neurobiological bases of empathy and compassion is closely linked to the field of contemplative neuroscience,^52^, ^53^, ^54^, ^55^, ^57^, ^58^, ^59^, ^150^ which includes the trainability of compassion and other qualities like mindfulness and associated plasticity in the brain and at the behavioral level. The next section introduces the field of contemplative neurosciences and key plasticity studies that have led to the differentiation between the neuronal networks underlying empathy and compassion.^51^ I then introduce key large‐scale longitudinal mental training studies focusing on the cultivation of attention‐based mindfulness, compassion, and other social capacities, such as the Shamata project,^17^ the Medit‐Aging study,^18^ and one of the largest multi‐method and multi‐disciplinary mental training studies on the social brain, the ReSource project (see details below),^19^ as the latter demonstrates how various mental practices differentially benefit brain plasticity, stress‐related hormonal and autonomic systems, and prosocial behaviors.^20^

### The emergence of the field of contemplative sciences

Around the time social neuroscience was advancing in understanding the neural components of the social mind, the field of contemplative science emerged, focusing on the trainability of positive qualities like mindfulness and compassion.^52^, ^53^, ^54^, ^55^, ^57^, ^58^, ^59^, ^151^ This field bridges ancient meditative practices, primarily from the East, with modern scientific inquiry.^151^, ^152^ Early initiatives led by the Mind and Life Institute, originally founded by neuroscientist Francisco Varela and the Dalai Lama in 1987, fostered dialogs between scientists and Buddhist scholars, paving the way for integrating Eastern and Western perspectives. These dialogs led to research grants and later the empirical investigation and development of meditation‐ and mindfulness‐based intervention programs, also summarized as MBIs, investigating the effects of contemplative practices on the brain, behavior, and well‐being.^53^, ^153^, ^154^

One prominent MBI program is the 8‐week Mindfulness‐Based Stress Reduction (MBSR) program by Kabat‐Zinn,^155^ followed by the mindfulness‐based cognitive therapy program.^156^ This wave of MBIs was followed by programs focusing more on the cultivation of socio‐affective capacities such as compassion, such as the 8‐week compassion cultivation training,^151^, ^157^ the mindfulness self‐compassion program by Christine Neff and Christopher Germer,^158^ and compassion‐focused therapy by Paul Gilbert.^85^, ^87^, ^88^, ^159^

Over the years, studies have confirmed the benefits of these MBIs and similar programs, showing improvements in stress reduction,^2^, ^10^, ^160^, ^161^, ^162^, ^163^ general well‐being,^164^, ^165^ mental health,^11^, ^166^, ^167^ social motivation and skills,^3^, ^4^, ^168^, ^169^ as well as prosocial and cooperative behaviors.^14^, ^15^, ^16^, ^170^

Researchers began exploring the philosophical and practical implications, advocating for a broader understanding of the mind's potential for positive change through contemplative practices and greater societal change through empirically informed public health and policy initiatives.^171^ These efforts have established contemplative science as an interdisciplinary field promoting well‐being through mindfulness and scientific rigor in different fields of society, ranging from health care and education to workplace well‐being.

### Training empathy and compassion

When neuroscientists started to become interested in contemplative sciences, their initial focus was mostly on understanding how different contemplative practices could alter brain structures of expert meditators, primarily Buddhist long‐term meditation practitioners, compared to novices, particularly with a focus on mindfulness and compassion practices.^172^, ^173^, ^174^, ^175^, ^176^ Research in this domain led to significant discoveries, including differentiating the neural underpinnings of empathy and compassion.^51^, ^90^, ^177^

Typically, in plasticity research, the investigation of brain plasticity starts with investigating the brains of experts—people who have practiced a specific skill for thousands of hours, such as musicians for motor plasticity or taxi drivers for spatial memory studies.^178^, ^179^, ^180^ Accordingly, in contemplative neuroscience, early studies explored the neuronal plasticity associated with cultivating mindfulness and compassion primarily by studying Buddhist monks or long‐term meditation practitioners with years or decades of regular practice engaging often daily in some types of compassion or loving‐kindness meditations.^172^, ^173^, ^174^, ^175^, ^176^ Richard Davidson's lab, for example, sought to identify the neural correlates of meditation through neuroimaging studies comparing novices to expert meditators, including Tibetan monks.^174^, ^175^, ^181^

Similarly, after having investigated the neuronal underpinnings of empathy and their modulating factors,^48^, ^130^, ^135^, ^136^ our lab got interested in exploring the malleability of these social emotions through explicit mental training and invited first a long‐term meditator, Buddhist monk, and former molecular biologist (Matthieu Ricard) to Rainer Goebel's laboratory, which had developed real‐time fMRI protocols to observe brain activation in real‐time during scanning,^182^ allowing the expert to engage in multiple different emotional (e.g., happiness, anger, disgust, compassion) mental states with different intensities (30%, 60%, or 90%) to observe how brain signals and networks can be modulated by willful mental production of different intensities of these inner states.^177^ When a long‐term practitioner is asked to engage in different compassion meditations while imagining, for example, the suffering of starving and neglected children in a mismanaged orphanage from a documentary seen the day before, activation in brain networks typically associated with reward and positive affect, such as the mPFC, nucleus accumbens, striatum, and mid insula,^183^, ^184^, ^185^ can be observed, rather than the well‐known empathy‐for‐pain networks illustrated in Figures 1 and 2A,B. Subjective reports confirmed that while generating compassion, the expert feels strongly altruistic with feelings of concern, love, and warmth, wishing for the children's relief from suffering rather than sharing their suffering. In contrast, when asked to only empathize with (resonate with) the children's pain, the typical empathy‐for‐pain network (AI/IFG, mACC) is activated; however, these empathic brain responses are now associated with high levels of reported empathic distress and negative affect,^186^, ^187^ and these negative feelings could only be transformed into more healthy states again when engaging in compassion. These unconventional experiments with a Buddhist monk are an example of the interdisciplinary nature of experiments done in contemplative science and at the same time lay the foundation for further research on a crucial distinction between two seemingly similar concepts, empathy and compassion, revealing that these two types of responses to the suffering of others rely on different neural networks.^51^, ^90^, ^187^

Furthermore, fMRI studies conducted in a larger group of expert long‐term meditation practitioners^49^ and non‐expert naive individuals^90^, ^177^ replicated the findings of distinct brain networks underlying empathy and compassion.^50^ For example, as illustrated in Figure 2B, naive participants who underwent empathy training showed increased activation in empathy‐for‐pain networks (AI, mACC) when viewing videos of people suffering, which was associated with increased negative affect (see Figure 2B, in blue). Conversely, participants who underwent compassion training showed activation in the compassion network (in contrast to an active control group learning a mnemonic technique), including the medial orbitofrontal cortex, ventral striatum, and pregenual anterior cingulate cortex (see Figure 2B, in red), areas that together form a network related to positive emotions,^184^, ^185^ affiliation and love,^188^, ^189^, ^190^ and reward.^183^, ^191^ In line with the qualitative accounts of Matthieu Ricard, this functional brain plasticity was accompanied by an increase in positive affect, and the increased negative affect after empathy training could be reduced back to baseline levels.^90^

As illustrated in Figure 2C, these findings led to a model distinguishing among empathy, empathic distress, and compassion on biological and psychological levels.^51^ Witnessing others’ suffering can trigger different emotional reactions. In untrained individuals, it typically activates a healthy empathic response (AI and mACC) associated with vicarious negative affect. However, excessive empathic activation can blur the important self–other distinctions outlined above, leading to empathic distress characterized by withdrawal or reactive behaviors, and if experienced over long‐term, potential burn‐out. In contrast, compassionate responses are linked to positive, resilient states, and altruistic motivation, promoting prosocial behaviors.^14^, ^15^ Compassion is suggested to rely on an important and evolutionarily old altruistic motivational system, the affiliative or care system.^192^, ^193^, ^194^, ^195^ Importantly, as much as in non‐trained people, empathy is typically the first response when exposed to the suffering of others before compassion arises; mental experts such as Matthieu Ricard can also engage in pure compassion directly without a preceding empathic response.

Due to a lack of differentiation and social neuroscience research at the time, the often‐used term *compassion fatigue*, originally introduced to describe the emotional and physical exhaustion that caregivers, particularly those in helping professions, can experience due to the chronic stress of caring for others, particularly those who are suffering or in distress,^196^, ^197^ can now be seen as a misnomer because, based on above research, compassion rather fosters resilience. Instead, “empathic distress fatigue” would more accurately describe emotional exhaustion from taking on others’ suffering.^198^ High burnout rates in professions like health care and education stem partly from empathic distress due to constant exposure to others’ suffering.^199^, ^200^ It is thus of utmost importance to use these findings to inform the design of specific mental practices and training programs, helping to differentiate healthy empathy from empathic distress and how to move from empathy or even empathic distress into compassion to foster resilience. And indeed, the discovery of brain plasticity supporting the improvement of important social skills such as empathy and compassion has been encouraging, as such training does not only promote prosocial behaviors^14^, ^15^, ^16^, ^201^ but also enhances positive affect and resilience^202^, ^203^ and fosters mental health by reducing stress.^204^

The first wave of research in the field of contemplative neuroscience was characterized by studying only a few outcome measures in one domain in a given study, and the duration of an intervention was mostly in the domain of some days or weeks (mostly the classic 8‐week MBI program length). In contrast, a second wave dared to engage in large‐scale mental training studies, taking a more holistic approach by studying multiple outcome measures and designing intervention programs with durations of 3–18 months. In the next section, I summarize the findings of three outstanding large‐scale mental training studies, namely, the 3‐month Shamata project,^205^ the 9‐month ReSource project,^19^ and the 18‐month Medit‐Aging European study^18^, ^206^, ^207^ to exemplify how the training of different types of mental skills can induce plasticity not only on the level of brain structure and function but also on the hormonal, autonomic, and behavioral level associated with increases in mental health and well‐being.

### Large‐scale mental training studies

#### Shamatha project

The Shamatha Project, spearheaded by Cliff Saron on the research side and B. Alan Wallace on the teacher side, was among the first comprehensive scientific studies in the field of contemplative sciences examining the effects of intensive meditation practice on psychological and physiological well‐being. Launched in 2007 at the Shambhala Mountain Center in Colorado, this courageous project followed 60 experienced meditators who underwent 3 months of rigorous Shamatha meditation training, focusing mostly on attention but also on some compassion‐based practices.^17^, ^205^ Researchers assessed various metrics, including attention, emotional regulation, and stress levels, using a combination of psychological tests, physiological measures, and subjective reports. The findings highlighted significant improvements in perceptual processes and sustained attention, emotional regulation, and socio‐emotional functioning and overall well‐being.^208^, ^209^, ^210^ Researchers also assessed epigenetic markers of telomere length and telomerase activity to explore potential effects of meditation on cellular aging.^208^ Telomeres are protective caps at the ends of chromosomes that shorten with age and stress. Longer telomeres are generally associated with better cellular health and longevity. Although results did not show significant increases in telomere length, intense meditation could increase telomerase activity, an enzyme that helps maintain telomere length, and thus suggests potential long‐term benefits for cellular aging and health from intensive meditation. However, these results are still disputed, as other studies found no changes in diverse telomere markers even after 9 months of intensive training.^211^, ^212^

These findings advanced the understanding of the mind–body connection and offered empirical support for the benefits of intense and sustained meditation practice performed in an in‐person retreat center over several months. However, this study did not utilize a randomized clinical trial design with active control groups and meditation‐naïve participants randomly assigned to a training and an active control group, but rather recruited mostly experienced meditation practitioners who were already deeply into Buddhist philosophy and practice and compared their effects to a wait‐list control group of a similar sample. Furthermore, the focus was not yet on investigating the differential effects of different types of practices on diverse outcome measures.

#### The ReSource project

**FIGURE 3:**
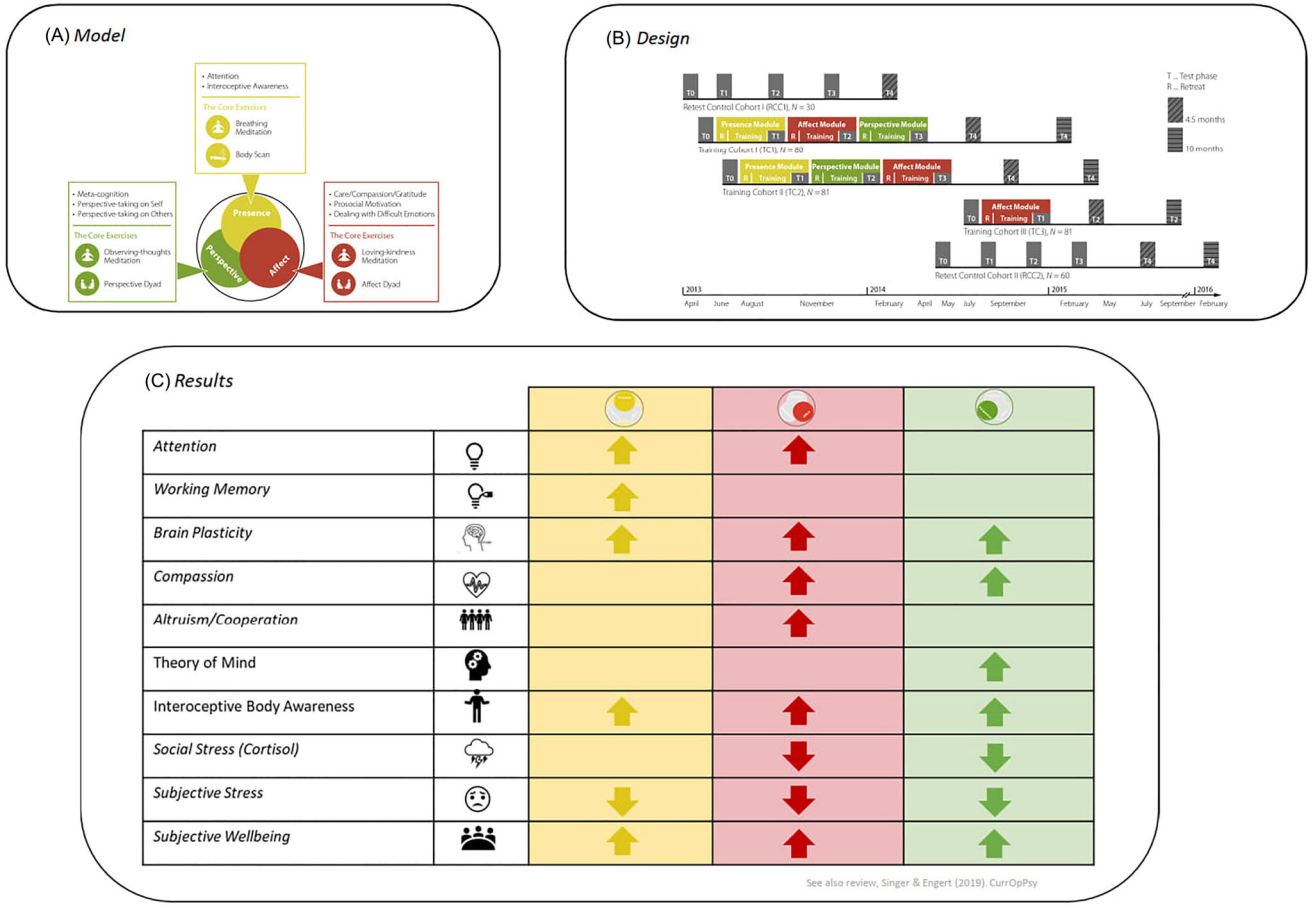
This figure depicts the model, design, and findings of the ReSource project, a large‐scale 9‐month longitudinal mental training study.^19^ Panel A illustrates the training module with three 3‐month training modules (presence, affect, perspective) and two daily core practices per module, which had to be practiced daily via an app. In addition, participants learned many mental practices in 3.5‐day retreats at the beginning of each module and in the 2 h weekly coaching sessions with teachers. Panel B illustrates the study design with its many measurement time points (*T*_0_–*T*_4_), with two main training cohorts serving as active control groups for each other (the order of the two social modules differs), an active affective control group to test specific effects of the presence module, and a re‐test control group. Panel C depicts the overall pattern of results showing module‐specific effects of different outcome variables. For details, see Ref. 20.

Inspired by the Shamatha project, the ReSource project^19^, ^20^ extended the duration of the intervention to 9 months and aimed to investigate the differential effects of various types of mental practices over three distinct 3‐month training modules in naïve participants. Unlike other interventions that blend different types of practices, the ReSource project focused on training specific skills in three different training modules, focusing on social brain skills, such as empathy, compassion, and cognitive perspective‐taking, and assessing their differential effects on a large battery of outcome measures, including functional and structural brain plasticity measures. As illustrated in Figure 3A, the three specific 3‐month modules were a mindfulness‐based attention module (presence), a socio‐emotional care‐ and compassion‐based module (affect), and a socio‐cognitive perspective‐taking module (perspective). Each module began with a 3‐day silent retreat, followed by weekly 2‐h teacher‐led in‐person sessions and daily practices via a custom app. The main cohorts performed the two social modules, affect and perspective, in different orders after the presence module, acting as active control groups to each other and enabling a comparative analysis of specific practice effects (see Figure 3B). A further active control group for the 3‐month presence modules, respectively, focused solely on a 3‐month affect module, and another 9‐month group that was only tested served as a retest control group to account for retest and seasonal effects. This design aimed to determine which type of contemplative practice impacts which domain, utilizing over 90 different markers, ranging from biological, hormonal, autonomic, and brain markers to behavioral tasks, subjective questionnaires, and phenomenological reports.^19^, ^20^

The study involved 332 meditation‐naive participants (age range = 18–55; mean age 40.74 years), primarily from Germany. Findings at present from over 50 publications from the ReSource project (see an overview in Figure 3C) demonstrated that daily mental practices over several months could induce plasticity at the brain, hormonal, and behavioral levels, enhancing even so‐called soft skills like empathy, compassion, and cognitive perspective‐taking across all ages and not only in younger cohorts. In addition, it revealed that it really matters what you practice, as the majority of findings revealed rather specific effects of each of the three training modules on diverse outcome measures.^20^

As Figure 1C illustrates, structural brain changes, measured through gray matter volume, revealed that different mental practice modules could induce gray matter plasticity in distinct brain networks.^4^ The presence module affected prefrontal areas linked to attention capacities.^213^ The affect module induced changes in parietal and fronto‐insular regions crucial for empathy and compassion.^51^, ^118^ The perspective module led to plasticity in the inferior frontal and temporo‐parietal junction, areas important for cognitive perspective‐taking (above ToM meta‐analyses here again). These structural changes correlated with behavioral improvements: increased ToM abilities post‐perspective training and enhanced compassion following affect training. Trautwein et al.^3^ further confirmed based on the EmpaTom task^118^ and a cue‐flanker attention task^214^ that indeed each of the three ReSource training modules also led to specific increases in these capacities, with attention increasing most after the presence module, compassion after the affect module, and ToM after the perspective module (see Figure 1C, lower part). These findings extended previous results showing structural brain plasticity after meditation practice^153^, ^154^ in revealing highly practice‐specific effects inducing grey matter plasticity in different neuronal networks of the social brain.

Such differential outcomes across the three modules were not limited to brain plasticity and social cognition but extended to various domains (for an overview of findings, see Figure 3C). For example, only the presence module improved working memory,^215^ specific aspects of interoceptive body awareness^216^, ^217^, ^218^, and reduced overall thoughts and mind wandering.^216^ The affect module, in contrast, uniquely boosted altruistic behaviors,^219^ acceptance, and positive thoughts and emotions of warmth and love.^93^, ^216^ The perspective module enhanced meta‐awareness,^216^ aligning with improved ToM abilities and related brain network changes.^3^ Notably, Engert et al.^2^ observed that cortisol responses to social stressors were significantly reduced only after the two social modules, affect and perspective. Interestingly, in contrast to the classic mindfulness‐based presence module, these social modules included a novel daily core practice, the contemplative dyad^21^, which is two partner‐based socio‐emotional (affect dyad) and socio‐cognitive (perspective dyad) exercises,^21^ I will discuss these in more detail below, as they seemed to be especially efficient in boosting social capacities and cohesion.

Another important aspect this 9‐month intervention study revealed is that some changes need time and cannot be achieved in just a few days or even weeks. Especially in the domain of training‐related changes in interoceptive body awareness, our ability to recognize and understand body signals, practicing 8–13 weeks of mindfulness (in the presence module) did not suffice to induce significant changes in a heart‐rate perception task or in an autonomic system bio‐feedback task, but it needed 6–9 months to achieve significant effects with small‐to‐medium effects sizes.^217^, ^220^ These findings point out the importance of maintaining daily training over longer periods and warn against the false promises of the mental health industry to achieve positive outcomes already after just a few minutes of app‐based meditations or short weekend courses.

#### The Medit‐Aging project

The ReSource project covered an age range from 18 to 55 years of age but did not yet focus on the effects of contemplative practices on older age groups. The investigation of the potential benefits of meditation on the aging process was the focus of a recent, now even 18‐month study, the Medit‐Aging study, led by Gaël Chételat in association with colleagues from diverse European labs.^18^, ^206^, ^207^ Also conducted over several years, researchers focus on assessing the impact of meditation on age‐related cognitive decline, emotional well‐being, and overall quality of life in 137 older meditation‐naive adults of 65+ years and older.

The findings from the Medit‐Aging study suggest that meditation and mindfulness practices can have significant positive impacts on the aging process. Thus, long‐term meditation practice was associated with the preservation of cognitive functions, including attention, memory, and executive function, which often decline with age.^206^, ^221^ As in previous studies,^4^, ^52^, ^53^, ^54^, ^57^, ^153^ changes in brain plasticity could even be observed in this older population,^207^ as well as better emotional regulation, reduced emotional reactivity, and increased resilience to stress, which are crucial for mental health in aging populations. In line with other studies, it seems as if contemplative practices can be considered a valuable intervention for promoting healthy aging and enhancing the quality of life in aging populations.^222^, ^223^, ^224^, ^225^

In summary, a second wave of large‐scale multi‐method longitudinal mental training studies demonstrated that MBIs and mental practices have indeed huge potential to not only foster mental health in general but also to boost adaptive and important social capacities such as empathy, compassion, and perspective‐taking on self and others associated with functional and structural plasticity in brain circuitries subserving these socio‐emotional or cognitive skills. Such plasticity is observed across a wide range of ages up to older age. Future studies will have to expand this research to earlier ages (into childhood and adolescence) to investigate the trainability of social skills in cohorts with much more plastic brains. The findings also highlight the importance of investigating the specific effects of different types of mental practices to allow the development of targeted interventions for different populations^20^ and the need for a better understanding of the mechanisms that underlie different types of mental practices, including novel ones that focus on directly targeting our ability to better relate to each other and train the relational brain by requiring practitioners to practice daily together with a partner.^21^ These findings also pave the way for broader, translational applications within society, urgently needed to provide solutions for the increasing levels of mental health problems and experienced isolation and loneliness, a problem that was exacerbated by the worldwide COVID‐19 pandemic.

## TRAINING THE RELATIONAL BRAIN

Having summarized evidence for distinct brain circuitries underlying social capacities like empathy, compassion, and perspective‐taking, and provided examples of how different mental practices can induce plasticity in the brain, autonomic, and endocrine systems, as well as enhance social skills and behaviors, I now turn to the investigation of the plasticity underlying our ability to relate to others and form meaningful and harmonious relationships through better listening skills or awareness of social conditionings and patterns. The next section will focus on novel intersubjective mental training approaches based on so‐called *dyads*, which, unlike previous MBIs involving mostly solitary practices done alone, involve others as practice partners. Specifically, I will explore novel dyadic training methods that hold promise for scalable online approaches to cultivating human relationships and social cohesion and discuss how these practices can help to reconnect with oneself, others, and humanity at large and overcome both social isolation and the polarization threatening the fabric of society.

### Relational mental practices: dyads and social connectedness

In psychology and communication, the term *dyads* refers broadly to interactions between two individuals. The origins of using dyad in this context can be traced to a few key sources, but the term itself originates from the Greek word *dyas*, meaning a pair or two. These interactions serve various purposes, such as strengthening relationships, studying communication patterns, and achieving therapeutic goals through role play.^226^, ^227^ The term *contemplative dyad* in the context of mental practices and contemplative traditions^21^ often refers to a structured, two‐person dialogue or meditation practice aimed at fostering deeper understanding, insight, or emotional connection.

As the name indicates, the specific form of contemplative dyads is deep interpersonal meditative practice where one person reflects on a topic, whereas the other listens actively without interrupting either verbally or non‐verbally.^21^ This format is used in many mindfulness programs, including Jon Kabat‐Zinn's MBSR, where open‐ended questions, the so‐called inquiry method, encourage self‐inquiry and self‐awareness during meditation.^155^, ^156^, ^228^, ^229^ Intensive retreat formats also utilize dyads, such as the Diamond Approach by A.H. Almaas,^230^ Gregory Kramer's Insight Dialogue,^231^, ^232^ and the Enlightenment Intensive retreats developed by Charles Berner in the late 1960s.^233^ These often involve long, partner‐based contemplative inquiries of 40 min or more with participants sitting face‐to‐face in a retreat setting.

In the context of the above‐mentioned ReSource project,^19^ researchers developed a science‐based skill‐learning approach differing from earlier in‐person retreat practices by requiring practitioners to practice dyads daily supported by an app in their everyday lives over weeks or months.^21^ These longer daily practice interventions aim to cultivate lasting social skills, such as empathy, perspective‐taking, and compassion toward self and others, as well as further inter‐relational skills such as a sense of shared humanity, tolerance, and becoming aware of social conditioning and schemata with a real person as a daily practice partner. Unlike traditional solitary practices that require maintaining internal images for compassion or loving‐kindness meditations,^165^, ^234^ this approach uses real‐time interactions with mostly unknown, weekly changing partners to build social skills and social cohesion and connectedness.

Although such contemplative dyad programs probably share some common features with psychotherapy—such as sharing emotional vulnerability in a safe space with a listening witness—it is important to note that contemplative dyad programs are not considered a therapy but a social form of mindfulness practice, with unknown naive partners being randomly paired weekly together to explore feelings, thoughts, and other inner states while another listens but never comments or responds in any way to what the other partner shares out loud during its exploration. It is, in other words, an aloud meditation done with a silent witness.

Typically, participants use a research app to engage in daily 12‐ to 15‐min dyad practices with randomly assigned partners. Each person reflects on two specific questions for 2.5 min each, practicing non‐judgmental listening. Partners change weekly to encourage a sense of shared humanity and tolerance. Depending on the questions, different skills can be trained. For example, in the ReSource project, two partner‐based practices, the affect dyad and the perspective dyad,^21^ were developed for the affect and perspective training modules, respectively. The affect dyad, for example, targets skills like accepting difficult emotions, better coping with stress, improving interoceptive body awareness, building resilience through gratitude, and enhancing social connectedness. Specifically, the listener asks, “describe a situation within the last 24 h in which you experienced a difficult emotion, and how did it feel in your body?” After 2.5 min, a gong signals the listener to ask, “describe a situation within the last 24 h in which you felt grateful, and how did that feel in your body?”. This second question often mitigates any negative affect arising from the first question. After answering both questions, the roles switch. In contrast, the perspective dyad focuses on learning to take different perspectives on one's own and others’ minds and belief systems. This practice involves learning aspects of the Internal Family Systems approach, which helps participants to identify and understand various “inner parts” of their psyche, thereby allowing them to develop a meta‐cognitive perspective on different personality aspects of themselves. The listener in the dyad enhances their ToM^3^ by trying to guess from which inner part and belief system the other is speaking (for more details, see Ref. 21).

More generally, compliance with the practice schedule is a critical topic in training research in general and in the contemplative sciences in particular, as many MBIs and other mental programs observe low or diminishing levels of adherence to daily practice over time, especially in the domain of mindfulness‐based apps. Introducing a novel daily practice with an unknown partner over many weeks may seem a risky choice for an intervention. However, surprisingly, results revealed that compliance with practicing daily dyads with partners was comparable to the practice frequency of required daily core meditation done alone at home^21^ or, in another more recent study, even higher than for classic mindfulness practice of the same length, especially when it came to voluntary continuation and sustainability of practice after the study had ended.^235^ Social norms of accountability may encourage compliance, as participants do not want to miss a scheduled appointment and let their partner down.

Furthermore, daily ratings before and after dyad practice indicated that participants enjoyed these daily partner‐based dyads, and their emotional well‐being improved afterward.^21^ They also reported feeling closer to their partner after both affect and perspective dyads. Interestingly, they felt even closer to their partner with each added training week, even if the matched partner was new and thus still unknown. The content they disclosed became more personal throughout the training, indicating growing connectedness and trust, even with always new partners. This suggests that a sense of social connectedness and shared humanity emerged through this inter‐relational practice.^236^ Despite the field still lacking scientific investigations of the effects of inter‐relational practices as compared to other solitary mental practices, these findings are in line with a few other studies using social practices but mostly focusing on the short‐term effects of a social meditation, mostly tested just after having practiced a dyad or even just mutually gazing at each other. Despite these studies not being based on skill‐learning approaches performed over several weeks or months, they also reveal feelings of increased social closeness or presence and empathy for the practice partner.^237^, ^238^

These newly developed intervention programs based on daily dyadic practices with a partner and supported by an app demonstrated high levels of practice compliance, probably outperforming classic mindfulness practices done alone, and significant positive outcomes in social connectedness, emotional disclosure to others, and other social qualities. These findings highlight the potential of dyadic practices to enhance social cohesion and social skills while reducing social stress, offering a promising approach for fostering deeper human connections and relationships, especially in a world increasingly suffering from polarization, social isolation, and loneliness—a trend exacerbated by the worldwide COVID‐19 pandemic.

### Addressing the mental health crisis through scalable online trainings

The world is grappling with rising mental health issues, with depression and anxiety being major contributors to global health burdens.^239^ Recent studies highlight a growing trend of increased loneliness, especially among young adults, who often report the highest levels of loneliness.^23^, ^240^ This loneliness epidemic^241^ has been exacerbated by the COVID‐19 pandemic.^242^, ^243^, ^244^ Other studies performed across the world during the pandemic also documented higher levels of depression, anxiety, and emotion regulation difficulties compared to pre‐pandemic levels.^245^, ^246^, ^247^, ^248^ Similarly, the CovSocial project^243^, ^244^, ^249^ confirmed in a large German sample that both lockdowns, but especially during the longer second lockdown with so‐called pandemic‐fatigue effects,^250^ came with increasing mental health burdens, including stress, depression, anxiety, and loneliness.^249^ Specifically, females, younger people, lower‐income groups, and individuals with previous clinical diagnoses and lower social belonging showed more vulnerable trajectories with higher levels of vulnerability and less resilient recovery profiles during prolonged stressors associated with multiple lockdowns^244^ a pattern observed also in other COVID‐19 studies worldwide.^251^, ^252^, ^253^, ^254^

#### Toward scalable app‐based, online interventions

These alarming mental health trends worldwide call for effective measures and interventions to mitigate mental health problems and loneliness on a large scale, focusing on scalable and easily accessible digital approaches. Indeed, the pandemic has led to a surge in mindfulness‐ and compassion‐based apps and online intervention programs,^255^, ^256^, ^257^ with several empirical studies confirming improvements in mental health following online mindfulness‐based and socio‐emotional interventions, with a focus on attention‐based breathing practices and training empathy and self‐compassion.^255^, ^258^, ^259^, ^260^, ^261^, ^262^ Overall, many of these online studies could replicate previous findings of earlier in‐person MBI research showing, for example, reduction in stress, including work stress,^263^ psychological distress, anxiety, and depression,^263^, ^264^, ^265^, ^266^ and improvements in mindfulness and different aspects of emotional well‐being.^264^, ^266^

Many of these studies, however, incorporate a mix of various mindfulness and socio‐emotional compassion‐focused practices simultaneously, making it difficult to both identify the specific effects of a given practice type as well as identify the underlying mechanisms and active ingredients of these different types of practices. Furthermore, to our knowledge, so far none of these studies have incorporated inter‐relational mindfulness practices such as contemplative dyads that represent particularly promising approaches to combat the loneliness epidemics.^241^

In the following section, I summarize recent findings from the CovSocial project's intervention phase,^22^ as a unique example so far of an online mental health intervention study that directly compared the effects of two app‐based online contemplative training programs, a mindfulness‐based and a socio‐emotional dyad intervention, and started exploring the underlying active ingredients and mechanisms, a line of inquiry that has been largely neglected in contemplative sciences so far, especially in the domain of socio‐emotional interventions (but see, for example, Refs. 267, 268, 269, 270).

#### The CovSocial project: comparing dyadic with mindfulness online trainings

**FIGURE 4:**
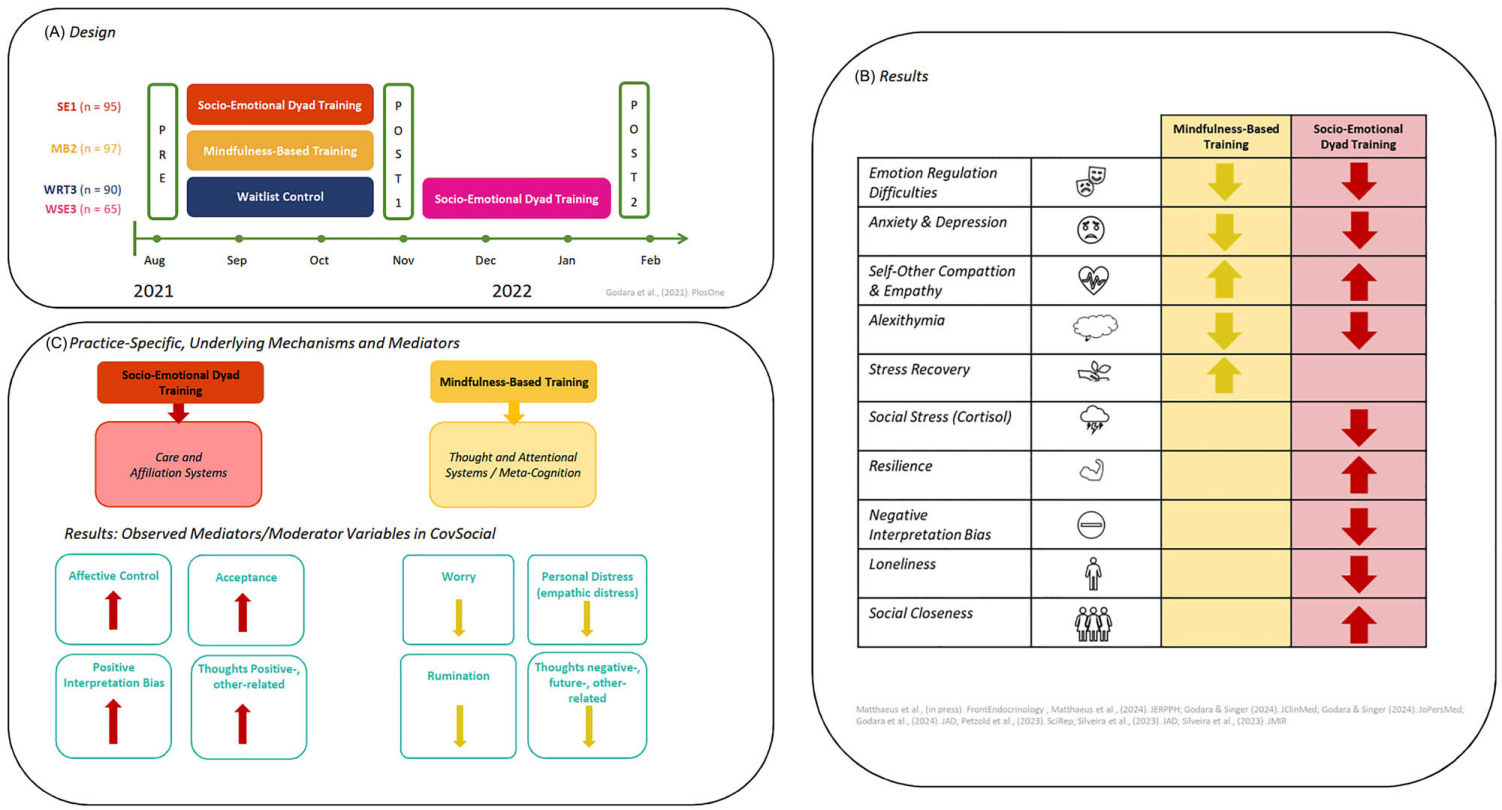
This figure depicts the study design, results, and mechanistic model resulting from the intervention phase 2 of the CovSocial project.^22^ Panel A illustrates the test design with pre‐ and posttest for the two training cohorts, a mindfulness (yellow) and a socio‐emotional dyad group (red), as well as the wait‐list control group (blue), which received an affect dyad training later on (pink). Panel B depicts a schematic overview of the common and differential results observed in this study for different outcome variables. Panel C depicts a tentative model suggesting differential underlying mechanisms and systems driving different types of practices, with thought, attentional, and meta‐cognitive processes underlying attention‐based mindfulness practices and affiliation and care‐based systems underlying relational partner‐based affective dyadic practices. The turquoise boxes below summarize observed findings from mediation and moderator analyses of the CovSocial project, showing on the bottom left for the dyad training group measures of affective control, positive interpretation bias, positive other‐related thoughts, and acceptance, and on the bottom right for the mindfulness group measures of worry, rumination, personal distress, and negative, future‐ and other‐related thoughts to be relevant variables in explaining observed training‐related changes in primary outcome measures.

The aim of the second phase of the CovSocial study, a longitudinal study that originally assessed mental health burdens, resilience, and social cohesion during the two lockdowns in a large community sample in Germany in 2020 and 2021,^22^, ^244^ was to counteract the detrimental effects on mental health declines observed with each passing month in lockdowns and to reduce loneliness, depression, and anxiety while boosting resilience, social cohesion, and connections through low‐dose, app‐based online mental training interventions. As illustrated in Figure 4A, the intervention study compared a 10‐week classic mindfulness program featuring 12 min of daily solitary, attention‐based meditation practices to a 10‐week socio‐emotional partner‐based dyad training. In the dyad training, participants practiced the affect dyad introduced already above daily with a partner, changing partners weekly as in the ReSource project.^19^ Such a design allowed for the first time to directly compare the effects of an inter‐relational dyadic practice with a classic mindfulness practice done alone but with the same daily intensity and teaching plan using the same app and with the same amount of weekly coaching sessions taught by the same teachers.

An important aspect of this 10‐week app‐based online intervention program was the inclusion of weekly 2‐h online coaching sessions led by expert dyad and mindfulness teachers. These sessions deepened practice through group sharing and short 15‐min talks on relevant topics. Effective practice of interpersonal, vulnerable partner‐based dyadic practices requires thorough onboarding to create a psychologically safe space and teach participants the practice's rules and structure. Although the affect dyad's two simple questions may seem straightforward, it is crucial to teach proper engagement, such as distinguishing gratitude from high‐arousal emotions and recognizing genuine body language. The 10 coaching sessions in the dyad intervention group covered topics such as body language and interoceptive awareness, how to listen empathically and regulate empathic distress, what is gratitude, and how to accept difficult emotions. They also addressed dyadic inter‐relational practices, such as recognizing social conditioning and patterns. Understanding social triggers and reactions is part of the learning experience in inter‐relational partner‐based practice, as dyad partners may be late, forget appointments, or discuss controversial topics, impacting the experience and relationship. This awareness thus directly trains relational capacities and underlying brain functions. A similar weekly 2‐h coaching journey covering attention‐based mindfulness topics was taught by the same teachers to the mindfulness intervention group.

As illustrated in Figure 4B, the results revealed an interesting pattern of common and unique effects from these purely online 10‐week daily practice interventions. In line with previous findings,^255^, ^263^, ^264^, ^265^, ^266^ both the mindfulness and dyad interventions reduced depression, anxiety, and emotion regulation difficulties, as assessed through well‐known validated questionnaires.^7^, ^167^ Both interventions also boosted body awareness, decreased alexithymia,^271^ and increased social capacities, such as empathy, self‐compassion, and compassion for others, measured by both questionnaires and a computer‐based task, the EmpaToM.^118^, ^168^

Interestingly, despite these shared effects, further analyses revealed unique effects of each practice as well as the insight that the underlying mechanisms driving observed improvements in mental health and prosocial capacities seem to differ between the two types of practices. Although classic solitary attention‐based mindfulness practice seems to act mostly on stabilizing and calming the mind and reducing rumination and negative thought patterns,^166^ the social–emotional dyad training seems to act more through activation of affiliative and care‐based motivational systems^2^, ^193^, ^194^, ^272^ and boost prosocial motivation, acceptance, and optimism.^2^, ^7^, ^219^ Specifically, reductions in depression and anxiety and increases in resilience were best predicted by weekly increases assessed over the 10 training weeks in acceptance and affective control in the dyadic group and by weekly reductions in rumination and worry in the mindfulness group.^166^ In line with these findings, only dyad training was able to boost resilience as measured through the CD‐RISC as well as positive interpretation bias as measured through the scrambled sentence task,^7^ along with positive thought patterns related to others,^273^ assessed with the cube of thought.^274^ In contrast, mindfulness practices could reduce active thoughts, in particular negative, future‐, other‐related, and negative thoughts, a finding that is in line with previous findings also showing an overall reduction in thinking processes after attention‐based mindfulness practices.^216^

#### Toward a mechanistic model

These findings give first indications that the mechanisms, that is, active psychological ingredients underlying these different types of practices, differ. The affect dyad seems to promote a positive outlook and optimism through a daily focus on gratitude and acceptance (see Figure 4C). This is supported by findings showing that increases in self‐compassion after dyad training were predicted by increases in acceptance assessed weekly as mechanisms every week throughout the 10‐week intervention.

Further analyses demonstrated more unique effects of the social training, which align well with the view that socio‐emotional dyad training works through activating affiliative‐ and care‐based motivational systems. Only partner‐based practices could reduce loneliness, as assessed both by the UCLA loneliness trait scale pre and post the 10‐week training and also with app‐based daily momentary assessment of experienced loneliness in your daily life.^235^ In line with previous findings of the ReSource project,^21^ practicing daily affect dyads could boost feelings of perceived closeness to the other practice partner just after a given 12‐min practice. Remarkably, the low‐dose online dyad training, but not the mindfulness training, significantly reduced the hormonal cortisol stress response to a social stressor,^275^ replicating earlier results showing that only training modules incorporating daily inter‐relational practices with a partner could reduce social stress on cortisol levels after experiencing a social stressor in the lab.^2^ Such findings suggest that empathic, non‐judgmental listening, exploring emotional vulnerabilities with a partner, and sharing intimate emotions can buffer against social stress, reducing fear of judgment—a major cause of social stress. It makes sense that mental practices not involving any other person in them, such as attention‐based mindfulness practices, are less efficient in reducing social stress elicited by social fears and anxieties of being judged or rejected.

These findings highlight the power of inter‐relational dyad practices in boosting social capacities, social connectedness, resilience, and a positive outlook on life while reducing social stress. Compared to solitary attention‐based mindfulness practices that seem to work on attentional and thought processes by calming the mind and reducing maladaptive ruminative and worry patterns, socio‐emotional dyad practices seem to rather work through activating care and affiliative systems, increasing acceptance and socio‐emotional regulation.

Such a view, as illustrated in Figure 4C, is in line with earlier proposals suggesting that key mechanisms of mindfulness may be underlying changes in cognitive capacities including working memory, executive functions and attention regulation, aspects of interoceptive body awareness, as well as aspects of self‐awareness, self‐regulation, and self‐transcendence, while reducing mind‐wandering.^215^, ^217^, ^267^, ^268^, ^276^, ^277^, ^278^ In contrast to mindfulness, scholars focusing on compassion‐based interventions and therapy^85^, ^158^ have suggested that compassion‐based intervention works mainly by individuals learning to approach their emotions with kindness, self‐acceptance and understanding, reducing emotional reactivity, improving emotion regulation, and enhancing resilience.^59^, ^279^, ^280^, ^281^, ^282^ Furthermore, by promoting positive social interactions and by these reducing feelings of isolation, they foster social connectedness, enhance social bonding, and reduce loneliness.^280^

Future studies should continue to work toward a deeper understanding of the active ingredients and mechanisms underlying the effects of diverse mental practices to allow the field to move toward a more mechanistic and, as a result, more individualized approach to mental health.

Given the high prevalence of mental health issues like loneliness, increasing social stress, and depressive symptoms in modern society,^23^, ^24^, ^240^, ^241^, ^251^ these results suggest that scalable intervention approaches based on interpersonal dyad practices could effectively address mental health problems and social isolation and social stress symptoms. These practices also boost optimism, resilience, social capacities, and social cohesion—qualities urgently needed in our increasingly isolated and polarized societies.

## CONCLUSION AND OUTSTANDING QUESTIONS

In this review, I have shown that the fields of social and contemplative neurosciences have demonstrated considerable progress in the past two decades, with their interaction allowing for the investigation of the plasticity of the social and relational brain. Although the first researchers started to dissect the social brain and identified distinct but interacting neuronal networks subserving our capacity to empathize, mentalize, and have compassion for another being, the second phase was marked by investigating the plasticity of these social skills, mostly through mindfulness‐ and compassion‐based or other mental training programs and interventions developed in the field of contemplative science. As more and more evidence is emerging that such mental training interventions do not only increase social capacities and induce brain plasticity in underlying neural networks but also boost various aspects of mental health and overall well‐being, the field has seen lately more and more efforts to scale and translate this research into different sectors of society to help build more resilience in populations with high risk of burn‐out such as healthcare workers or teachers. Furthermore, by exploring neural plasticity as a mechanism and developing new evidence‐based mental training programs, modern social and contemplative neurosciences are uniquely poised to offer creative approaches and innovative solutions remediating the pervasive epidemic of loneliness and disconnection eroding societal health and well‐being. One particularly promising example is a new line of interventions and associated research focusing on novel relational mental training approaches done with partners, so‐called contemplative dyads, also referred to as a new field of inter‐relational/intersubjective/or social mindfulness/meditation.

Such a social mental training approach could offer scalable solutions to counteract the increasing mental health problems and loneliness epidemic affecting more and more of our youngest cohorts. As research on such new relational mental practice formats is just emerging, future research would profit from comparing the effects of different types of dyadic mental practices adapted to increase different relational and social capacities, such as perspective‐taking, self‐compassion, or overcoming shame with each other as well as with more classic mindfulness‐based approaches and programs. Finally, future research will have to further determine the psychological processes underlying dyadic practices and help identify different active ingredients such as effects of social presence and social support of another being and the effects elicited by the specific practice contents (e.g., dyad questions posed).

Further, future plasticity research in the domain of social and contemplative neurosciences will have to continue investigating the underlying mechanisms and neuronal processes driving the effects of different mental practices to better identify who may most profit from which training and to work toward individualized approaches. So far, no brain imaging study has, for example, been performed exploring the neuronal processes underlying the novel inter‐relational dyadic practices, and it would certainly be interesting to compare how these practices go beyond activating the so‐far well‐known social brain networks underlying empathy, compassion, or ToM, perhaps adding circuitries involved in human attachment, trust, and cooperation.^193^, ^194^, ^283^ Furthermore, the trainability of different types of listening skills with relational practices, such as empathic as compared to compassionate listening, and their differential effects on mental health or team climate, have not been investigated so far.

Similarly, a person‐specific approach would benefit from further clinical research investigating the suitability of different mental training programs and practices for different patient populations.

Overall, most mental intervention studies based on MBIs or similar contemplative protocols have been focusing on assessing their effects right before and after a given 8‐week or several month‐long intervention, mostly ignoring, however, the long‐term effects of such training over longer timespans, both when participants continue or discontinue their regular practice. Similar to the domains of sport, it is known that observed changes in the brain after practice can revert back without practice,^284^, ^285^, ^286^, ^287^ and that to reach sustainable levels of improved mental health it is very likely that many beneficial effects only show up over longer durations of regular practice (see, e.g., Ref. 217). Future studies will need to extend testing to months or even years after the initial mental interventions end and give participants the opportunity to further practice freely to investigate dose‐dependent, as well as long‐term, effects of MBIs.

Finally, the focus so far has mostly been on studying the effects of such intervention on changes in an individual, be they changes in the brain, behavior, hormones, or well‐being of an individual. To understand how such individual change affects larger system changes, be it in hospitals, schools, or other work environments, it will be necessary to develop new paradigms enabling the assessment of changes in larger groups or even institutions and nations. Thus, still much research is needed to address the problem of how training the brains of individuals can address major societal problems such as the climate crisis, poverty during plenty, or the erosion of democratic systems. It will need the development of new translational approaches and measures that enable linkage of individual change to system change measures and require larger samples and longer study durations to assess long‐term effects. Such translational approaches may in the future lead to the emergence of another field—by analogy to other translational sciences such as translational medicine^288^, ^289^, ^290^, ^291^—called *translational social neuroscience*. With such efforts, the insights gained when studying the foundations of the social and relational brain and its plasticity could be translated to support different sectors of society in urgent need of intervention approaches and programs buffering the adverse effects of the growing mental crisis by boosting resilience, mental health, and social cohesion.

## CONFLICT OF INTEREST STATEMENT

The author declares no conflicts of interest.

### PEER REVIEW

The peer review history for this article is available at https://publons.com/publon/10.1111/nyas.15319.
